# Public activities preceding the onset of acute respiratory infection syndromes in adults in England - implications for the use of social distancing to control pandemic respiratory infections.

**DOI:** 10.12688/wellcomeopenres.15795.1

**Published:** 2020-03-30

**Authors:** Andrew C. Hayward, Sarah Beale, Anne M. Johnson, Ellen B. Fragaszy, Flu Watch Group

**Affiliations:** 1 UCL Research Department of Epidemiology & Public Health, UCL, London, WC1E 7HB, UK; 2 UCL Public Health Data Science Research Group, Institute of Health Informatics, UCL, London, NW1 2DA, UK; 3 UCL Institute of Global Health, UCL, London, WC1E 6JB, UK; 4 Department of Infectious Disease Epidemiology, London School of Hygiene and Tropical Medicine, London, WC1E 7HT, UK

**Keywords:** Acute Respiratory Infection, Pandemic, Transmission, Social Distancing, COVID-19

## Abstract

**Background: **Social distancing measures may reduce the spread of emerging respiratory infections however, there is little empirical data on how exposure to crowded places affects risk of acute respiratory infection.

**Methods: **We used a case-crossover design nested in a community cohort to compare self-reported measures of activities during the week before infection onset and baseline periods. The design eliminates the effect of non-time-varying confounders. Time-varying confounders were addressed by exclusion of illnesses around the Christmas period and seasonal adjustment.

**Results: **626 participants had paired data from the week before 1005 illnesses and the week before baseline. Each additional day of undertaking the following activities in the prior week was associated with illness onset: Spending more than five minutes in a room with someone (other than a household member) who has a cold (Seasonally adjusted OR 1·15, *p*=0·003); use of underground trains (1·31, *p*=0·036); use of supermarkets (1·32, *p*<0·001); attending a theatre, cinema or concert (1·26, *p*=0·032); eating out at a café, restaurant or canteen (1·25, *p*=0·003); and attending parties (1·47, *p*<0·001). Undertaking the following activities at least once in the previous week was associated with illness onset: using a bus, (aOR 1.48, p=0.049), shopping at small shops (1.9, p<0.002) attending a place of worship (1.81, p=0.005).

**Conclusions: **Exposure to potentially crowded places, public transport and to individuals with a cold increases risk of acquiring circulating acute respiratory infections. This suggests social distancing measures can have an important impact on slowing transmission of emerging respiratory infections.

## Introduction

The emergence of COVID-19 has led governments and public health agencies around the world to recommend social distancing measures in an attempt to contain the epidemic ^1, 2^. Identification of cases and isolation, either in quarantine facilities or at home, is a key strategic measure in the containment phase. As the infection becomes more widespread with established community transmission, measures to reduce exposure to crowded places may be recommended in an attempt to delay further spread and reduce peaks in healthcare activity. Intensive social distance measures in Wuhan, China appear to have helped to control the outbreak, at least in the short-term ^3^. Although there is evidence to support the use of hygienic measures such as hand hygiene, empirical evidence to support the use of social distancing is very limited ^4^. In the context of pandemic influenza, modelling studies suggest a role for social distancing in combination with other strategies ^5^ but, at least in the context of the 2009 influenza pandemic, where transmission was high but case fatality was low, social distancing measures were likely not to have been cost effective ^6^. Qualitative research shows that hand and respiratory hygiene were viewed as familiar and socially responsible actions to take but that there is ambivalence about adopting isolation and personal distancing behaviours due to their perceived adverse impact and potential to attract social stigma ^7^. In the context of COVID-19, which appears to transmit readily and to have an appreciably higher case fatality than H1N1pdm2009 ^8^, social distancing measures maybe warranted to slow the spread of the infection.

Respiratory infections transmit through contact and airborne routes. In contact transmission self- inoculation of mucous membranes by contaminated hands may involve direct transfer of virus from one infected person to another or indirect transfer of virus through contaminated intermediate objects (fomites). Airborne transmission can involve droplets (>5 μm) which can lead to short range transmission through direct deposition of droplets on mucous membranes and the upper respiratory tract through coughing, sneezing and breathing of the infected individual and aerosol transmission with droplet nuclei which can remain suspended in the air for long periods and be inhaled and deposited along the respiratory tract, including the lower airways ^9^. Assessing the relative importance of these modalities in respiratory infection transmission is complex. The relative importance may also vary by setting and pathogen. In the context of crowded public spaces rather than direct contact with infected individuals indirect contact and aerosol transmission may become relatively more important than in the context of face to face contact with infected individuals. Understanding the extent to which exposure to potentially crowded public places increases the risk of acquiring common respiratory infections, could help to inform control of emerging respiratory infections, which likely transmit using similar modalities.We aimed to assess this through analysis of the Flu Watch study ^10^, a large community cohort study that was designed to investigate incidence and risk factors for influenza.

## Methods

### Study design and procedure

Flu Watch was a community cohort study of respiratory infection occurrence and risk factors which followed up households across England and Wales through the winter seasons of 2006/7–2010/10 ^10, 11^. Participants were randomly selected from patient lists of general practitioners and sent letters to recruit the whole household. Those with terminal illness or who were participating in other research studies were excluded. Households were recruited in the autumn and followed up through winter periods until spring. During follow up, participants were contacted weekly and asked to report whether they had a cough, cold, sore throat or fever. They were also asked to complete illness diaries recording symptoms for any acute respiratory illness. In this study we are utilising self-reported respiratory infection regardless of whether or not the infection was laboratory confirmed. During the first three years of Flu Watch (2006/7, 2007/8, 2008/9) illness diaries included a series of questions on how many days in the week before illness onset a series of activities had occurred. These activities were: use of bus, underground train, train, plane, public house/bar/nightclub, eating out at a restaurant/café/canteen, going to a place of worship, going to a party for adults, going to the supermarket or small shops, going to the cinema/theatre/sports event, and playing sport., The same questions were asked at baseline. We also asked whether participants had spent more than five minutes in a room with anybody with a cold other than a member of their own household.

We hypothesised that higher levels of exposure to the public in venues such as public transport, supermarkets, cinemas etc. and contact with those with the common cold outside the household would increase the likelihood of infection, and that such activities would therefore be more common in the week before onset of an acute respiratory infection than at baseline. We used a case-crossover design ^12^, in which cases act as their own controls during a non-exposure time-period (i.e. baseline in the present study), to investigate whether exposure to the public in venues such as public transport, supermarkets, cinemas etc. and contact with those with the common cold were associated with acute respiratory infection syndromes. Case-crossover designs are appropriate to investigate the effect of transient exposures (e.g. exposure to infections in public places) on an acute outcome (e.g. acute respiratory infection syndrome) within a plausible hazard period (e.g. one week, to account for the 1–7 day incubation period of commonly circulating respiratory pathogens ^13^). As this is a paired test, comparing individuals in the week before baseline (referent period) with themselves in the week before illness onset (hazard period), non-time varying variables cannot act as confounders.

### Analysis

Analyses used Stata v 15. Each activity variable was recoded to a binary variable as to whether or not the activity occurred at least once during the week. Since we asked about different modes of public transport separately, we assigned no use of public transport as the baseline variable in each separate mode of transport analysis. Stata *mi logit* commands were used to impute missing data for binary exposure variables (activity conducted during week vs activity not conducted) using season, age group, gender, and illness vs baseline as predictors. We did not attempt to impute the frequency of exposure (0/7 days). In the UK context, social activities such as going to parties, meals out, or going out to public houses, bars or nightclubs increase in the period before Christmas and New Year, so, for these activities we excluded illnesses occurring in weeks 50 to week 1 (equating to exposures in week 49–52). Attendance at a place of worship is also more common for Christmas related services so we excluded illnesses in weeks 51 and 52 (equating to exposures in week 50 and 51) for the ‘visiting a place of worship’ activity.

As recommended for the analysis of case-crossover designs ^12, 14^, we used conditional logistic regression to analyse paired data and control for season (winter period October-March, Spring/Summer period April-September). Models were adjusted for lack of independence (multiple illnesses per participant) using robust standard errors (Stata cluster command). For binary (imputed) exposures these analyses were conducted using Stata mi commands. The analyses were then repeated on unimputed data treating the exposures as continuous variables (0–7 days) to assess the impact of increasing frequency of exposure.

### Ethics and consent

The protocol was approved by the Oxford Multi-Centre Research Ethics Committee (06/Q1604/103). Written consent was obtained by post.

## Results

626 participants reported at least one acute respiratory infection syndrome during follow up (1005 illnesses in total) and had paired pre-baseline and pre-illness follow up (see underlying data ^15^).

Table 1 shows the characteristics of the 626 participants with paired baseline and illness diaries. The majority of participants were aged 16–64 years (65·18%) and were of White British ethnicity (96·78%). Approximately one third had chronic illness (30·67%).

**Table 1.** Baseline characteristics of participants with paired pre-baseline and pre-illness activity records.

	Category	*n*	%
Age	0–4	45	7·19
	5-15	96	15·34
	Age 16–64	408	65·18
	Age 65+	77	12·30
Gender	Male	281	44·89
	Female	345	55·11
Winter season	2006/7	214	34·19
	2007/8	283	45·21
	2008/9	129	20·61
Ethnicity	White British	571	96·78
	Other	11	3·22
Long-term illness	Yes	192	30·67
	No	434	69·33

Table 2 shows the proportion of those reporting each activity in the week before baseline and the week before illness (proportions based on imputed data – missing data accounted for between 0·26% and 7·57% of observations). Unadjusted and seasonally-adjusted odds ratios, 95% confidence intervals and *p*-values are shown. 

**Table 2.** Comparison of exposures in week before onset of acute respiratory infection and week before baseline survey.

Risk Factor	Risk factor in week before baseline- n (%)	Risk factor in week before illness – n (%)	Paired OR (95% CI) and seasonally adjusted OR (95% CI)	*P-*value (adjusted *p-*value)
Travelled by bus *	175 17·41%	207 20·64%	1·56 (1·06-2·29) 1·48 (1·00-2·19)	0·024 0·049
Travelled by train *	106 (10·55%)	109 (10·83%)	1·06 (0·67-1·66) 1·06 (0·67-1·67)	0·798 0·794
Travelled by underground train *	42 (4·18%)	52 (5·14%)	1·51 (0·77-2·94) 1·84 (0·96-3·51)	0·227 0·066
Travelled by plane *	32 (3·18%)	28 (2·80%)	0·86 (0·42-1·60) 0·91 (0·48-1·71)	0·635 0·764
Had contact with somebody with a cold **	285 (39·67%)	370 (51·36%)	1·79 (1·36-2·36) 2·02 (1·52-2·71)	<0·001 <0·001
Played a team sport **	86 (11·98%)	90 (12·47%)	1·09 (0·62-1·92) 1·22 (0·68-2·19)	0·757 0·494
Went to the theatre, Cinema or sports event **	154 (21·39%)	178 (24·73)	1·14 (0·82-1·59) 1·41 (0·82-2·42)	0·430 0·216
Went to the supermarket **	622 (86·36%)	650 (90·27%)	1·71 (1·09-2·69) 1·75 (1·09-2·81)	0·020 0·019
Went to small shops **	570 (79·17%)	614 (85·23%)	1·89 (1·27-2·80) 1·90 (1·26-2·85)	0·002 0·002
Went to pub, bar or nightclub ***	258 (42·77%)	267 (44·25%)	1·24 (0·87-1·77) 1·24 (0·86-1·79)	0·242 0·253
Went to a café, restaurant or canteen for a meal ***	303v (50·32%)	338 (56·07%)	1·39 (1·03-1·89) 1·65 (1·20-2·26)	0·033 0·002
Went to a party mainly for adults ***	101 (16·68%)	131 (21·67%)	1·53 (1·07-2·19) 1·49 (1·03-2.17)	0·018 0·036
Went to a place of worship ***	121 (16·44%)	149 (20·24%)	1·73 (1·08-2·78) 1·81 (1·08-3·04)	0·022 0·005

Numbers, percentages and analyses are based on imputed data (missing data accounted for between 0·26% and 7·57% of exposure observations) * Data from all ages -number of illnesses ( *n*) – 1005, **Data from adults aged 16 years or above – number of illnesses remaining – 720, ***excludes activities in weeks 50 to week 1 (inclusive) when social activities are increased due to celebrations around Christmas and New Year – number of illnesses remaining - 603, ****excludes activities in week 51 and 52 (inclusive) when Christmas church services occur – number of illnesses remaining – 736

Spending more than five minutes in the same room as someone with a cold (adjusted odds ratio [AOR] 2·02, 95% confidence interval [CI] 1·52-2·71, *p*183;001), travelling on a bus (AOR 1·48, 95% CI 1·00-2·19, *p=*0·049), shopping at a supermarket (AOR 1·75, 95% CI 1·09-2·81, *p=* 0·019) or at small shops (AOR 1·90, 95% CI 1·26-2·85, *p=*0·002), eating out at a restaurant, café or canteen (AOR 1·65, 95% CI 1·20-2·26, *p=*0·002), going to a party (AOR 1·49, 95% CI 1·03-2·17, *p*= 0·036) or place of worship (AOR 1·81, 95% CI 1·08-3·04, *p=*0·005) were more common in the week before illness than the baseline week. There were no significant associations with the less frequently used transportation modes (train, underground train and plane), or with playing team sports, visiting public houses, bars or nightclubs or going to the cinema or theatre.

Further analyses using the original 0–7 days per week classification of exposure frequencies as continuous variables within conditional logistic regression analyses (unimputed data) found that each additional day of exposure was associated with increased illness risk for: contact with someone with a cold (AOR for each day increase in exposure - 1·15, 95% CI 1·05-1·26, *p*=0·003); use of underground trains (AOR 1·31, 95% CI 1·02-1·69, *p*=0·036); use of supermarkets (AOR 1·32, 95% CI 1·16-1·49, *p*<·001); attending a theatre, cinema or concert (AOR 1·26, 95% CI 1·02-1·55, *p*=0·032); eating out at a café, restaurant or canteen (AOR 1·25, 95% CI 1·08-1·45, *p*=0·003); and attending parties (AOR 1·47, 95% CI 1·17-1·84, *p*<0·001).

## Discussion

This is the first study to investigate the impact of specific public activities on the risk of acquiring respiratory tract infection in a population-based cohort. Being in a room with a person with a cold, from outside the household for more than five minutes, markedly increases the likelihood of contracting an acute respiratory illness. Exposure to a wide range of potentially crowded settings also increases likelihood of illness including buses, underground trains, supermarkets and small shops, entertainment venues, eat-in food venues, drinking venues, parties and places of worship. 

Results corroborate previous literature linking greater frequency and duration of social activity (non-specific) with risk of influenza ^16, 17^ or influenza-like-illness ^18^. Only one previous cross-sectional study of Japanese older adults has examined the effect of specific social activities, i.e. group-based leisure activities, finding a relationship with self-reported influenza infection in women ^19^. The present study characterised the risk of a range of daily public activities relevant to infection spread in community settings.

Previous literature around the effects of transport on risk of acquiring respiratory tract infections has yielded mixed results. Use of public transport has been linked with increased risk of influenza ^20– 22^ and influenza-like-illness ^23, 24^ in some studies, while other found no such effect and low risk of transmission ^25, 26^.

Our study was based on large numbers of acute respiratory infection syndromes identified through active surveillance of adults. By measuring exposures in the week before illness we focussed on the likely incubation period of common respiratory infections. Exposures were recorded close to the time of occurrence, minimizing recall bias. By comparing exposures prior to periods of illness and at baseline in the same individuals we eliminated the impact of non-time-varying confounders. Our findings are robust to adjustment for time of year (season) and, for exposures that are strongly associated with the Christmas period, to removal of illnesses occurring in the run up to Christmas and New Year. We cannot exclude that our findings may be influenced by time-dependent variations in activities and levels of respiratory illness that are not accounted for by these measures, but note that our findings are consistent across a wide range of plausible public activities. The finding in relation to contact with somebody with a cold may be influenced by differential recall bias whereby those with a respiratory infection are more likely to remember such contact than those who are not ill. These findings relate to undifferentiated acute respiratory infections that are widespread during the winter. Common seasonal respiratory viruses and COVID-19 appear to be transmitted in similar ways i.e. via a combination of droplet, and direct and indirect contact with infected secretions and an uncertain amount of aerosol-based transmission ^27– 29^. Exposure to public places could increase each of these forms of exposure, for example through holding onto/touching surfaces, close contact with symptomatic people and inhalation of aerosols with viral particles.

In the context of widespread transmission of an emerging respiratory infection with appreciable mortality and morbidity (such as COVID-19) these findings support the need for people with symptoms of respiratory infection to minimise contact with others, for example through self-isolation or working from home. Minimising public activities such as use of public transport, shopping, eating out, attending entertainment venues, going to parties and places of worship may be helpful in slowing the spread of infection and reducing peak intensity of transmission and associated healthcare usage. Minimising exposure to public venues may be of particular relevance for the elderly and those with chronic illness who are more likely than healthy young adults to develop complications of infection and to die. The impact of reducing contact with crowds on quality of life, personal autonomy, livelihoods and the wider economy need to be balanced with the potential reduction in attack rates and the potential to reduce surges in healthcare activity and mortality.

## Data availability

### Underlying data

Open Science Framework: Public activities preceding the onset of acute respiratory infection syndromes in adults in England - implications for the use of social distancing to control pandemic respiratory infections. https://doi.org/10.17605/OSF.IO/84EVG ^15^

This project contains the following underlying data:

- Publicspacesrespdataarchive.dta (Public venue exposure data from Flu Watch, .dta format)
- Publicspacesrespdataarchive.csv (Public venue exposure data from Flu Watch, .csv format)

Data are available under the terms of the Creative Commons Zero "No rights reserved" data waiver (CC0 1.0 Public domain dedication).
