# Supplementary material for: Public activities preceding the onset of acute respiratory infection syndromes in adults in England - implications for the use of social distancing to control pandemic respiratory infections
Source: Wellcome Open Res. 2020 Mar 30;5:54. [Version 1] doi: 10.12688/wellcomeopenres.15795.1 (PMC7194223; doi:10.12688/wellcomeopenres.15795.1)
Supplement: Peer review history

# Peer review: Reviewer response for version 1

**DOI:** 10.21956/wellcomeopenres.17321.r38462

**Published:** 2020-04-30

**Authors:** Amra Uzicanin

**Affiliations:** 1 Community Interventions for Infection Control Unit, Division of Global Migration and Quarantine, Centers for Disease Control and Prevention, Atlanta, GA, USA

To assess the extent to which the exposure to crowded public places increases the risk of acquiring common respiratory infections, the authors performed a secondary analysis of the data from a large community cohort study designed to investigate incidence and risk factors for seasonal influenza implemented over a 3-year period (2006/7, 2007/8, 2008/9). The present analysis is concurrent with the initial wave of the SARS-CoV2 pandemic, during which many countries resorted to using the social distancing measures of previously unseen intensity, duration and geographic scope, most notably nationwide “lockdowns”, in an effort to avert complete collapse of health care infrastructure due to rapidly growing numbers of severely ill cases requiring prolonged hospital stays including intensive care. This paper reports the unique data on pre-pandemic exposure patterns to crowded public settings including local and long-range public transport, retail and restaurant facilities, sporting and art venues, places of worship and private functions. The case-crossover design is used to examine association between frequency and types of crowded settings attended, and a subsequent episode of self-reported infectious respiratory illness. As the SARS-CoV2 transmission will likely persist in large swaths of the globe well beyond the initial pandemic wave, results of this analysis are an important first step to better characterize the risk associated with frequenting crowded community settings and inform future public health strategies.

 Minor comments:

1. Have any of the self-reported respiratory illness episodes been laboratory-confirmed, even if only for a subset of study participants? If so, it would be helpful to include the illness etiology data for context.
2. Last paragraph in the Conclusions: consider changing “people with symptoms” to “all people”, because asymptomatic/pre-symptomatic individuals can transmit SARS-CoV2. Also, currently available data suggest that a substantial proportion of SARS-CoV2 infections are asymptomatic or pauci-symptomatic.

Is the work clearly and accurately presented and does it cite the current literature?

Yes

If applicable, is the statistical analysis and its interpretation appropriate?

I cannot comment. A qualified statistician is required.

Are all the source data underlying the results available to ensure full reproducibility?

Yes

Is the study design appropriate and is the work technically sound?

Yes

Are the conclusions drawn adequately supported by the results?

Yes

Are sufficient details of methods and analysis provided to allow replication by others?

Yes

Reviewer Expertise:

NA

I confirm that I have read this submission and believe that I have an appropriate level of expertise to confirm that it is of an acceptable scientific standard.

# Peer review: Reviewer response for version 1

**DOI:** 10.21956/wellcomeopenres.17321.r38380

**Published:** 2020-04-14

**Authors:** Benjamin J. Cowling

**Affiliations:** 1 WHO Collaborating Centre for Infectious Disease Epidemiology and Control, School of Public Health, Li Ka Shing Faculty of Medicine, The University of Hong Kong, Hong Kong, China

The authors have done a secondary analysis of the FluWatch data to examine the association of social contacts with risk of respiratory virus infections. They find that exposure to potentially crowded places, public transport and to individuals with a cold increases risk of acquiring circulating acute respiratory infections. This suggests social distancing measures can have an important impact on slowing transmission of emerging respiratory infections. The findings are important because social distancing is one of the most commonly used interventions against COVID-19, ranging from voluntary changes in behaviour right up to the mandatory lockdowns or “shelter-in-place” used in many countries in February, March and April 2020. What is now uncertain is the extent to which more modest social distancing strategies could achieve the objective of suppressing COVID-19 transmission.

 Minor comments:

1. Did you examine any specific respiratory viral infections such as influenza, or any known virus? If so these results would also be of interest. Please clarify this in the text.
2. In the first sentence of your Discussion section, you could consider changing "impact" to "association"?

Is the work clearly and accurately presented and does it cite the current literature?

Yes

If applicable, is the statistical analysis and its interpretation appropriate?

Yes

Are all the source data underlying the results available to ensure full reproducibility?

Yes

Is the study design appropriate and is the work technically sound?

Yes

Are the conclusions drawn adequately supported by the results?

Yes

Are sufficient details of methods and analysis provided to allow replication by others?

Yes

Reviewer Expertise:

Infectious disease epidemiology.

I confirm that I have read this submission and believe that I have an appropriate level of expertise to confirm that it is of an acceptable scientific standard.
